# lncRNA028466 regulates Th1/Th2 cytokine expression and associates with *Echinococcus granulosus* antigen P29 immunity

**DOI:** 10.1186/s13071-021-04795-2

**Published:** 2021-06-03

**Authors:** Chan Wang, Song-Hao Yang, Nan Niu, Jia Tao, Xian-Cai Du, Ji-Hui Yang, Ming-Xing Zhu, Ya-Na Wang, Wei Zhao

**Affiliations:** 1 Department of Medical genetics and Cell biology, School of Basic Medical Science of Ningxia Medical University, Yinchuan, 750004 Ningxia Hui Autonomous Region People’s Republic of China; 2 Center of Scientific Technology of Ningxia Medical University, Yinchuan, 750004 Ningxia Hui Autonomous Region People’s Republic of China; 3 Ningxia Key Laboratory of Prevention and Control of Common Infectious Diseases of Ningxia Medical University, Yinchuan, 750004 Ningxia Hui Autonomous Region People’s Republic of China

**Keywords:** *Echinococcus granulosus*, Long noncoding RNA 028466, *Echinococcus granulosus* antigen P29, CD4^+^ T cell, Cytokines

## Abstract

**Background:**

Cystic echinococcosis (CE) is a parasitic disease that is caused by *Echinococcus granulosus* (Eg)*.* The recombinant *Echinococcus granulosus* antigen P29 (r*Eg*.P29) was shown to confer effective immunity to sheep and mice during *E. granulosus* secondary infection in our previous study. In this study, we sought to investigate the ability of long noncoding RNA 028466 (lncRNA028466) as a regulator for the protective immunity mediated by r*Eg*.P29 vaccination and to study the effects of lncRNA028466 on CD4^+^T cell differentiation in mice spleen.

**Methods:**

Female BALB/c mice were divided into two groups and were vaccinated subcutaneously with r*Eg*.P29 antigen and PBS as a control (12 mice each group). Following prime-boost vaccination, CD4^+^T, CD8^+^T, and B cells from the spleen were isolated by flow cytometry. Quantitative real-time PCR (qRT-PCR) was performed to measure the expression of lncRNA028466 in these three kinds of cells. Then, lncRNA028466 was overexpressed and knocked down in naive CD4^+^T cells, and Th1 and Th2 cytokine expression was detected. qRT-PCR, western blot, and ELISA were performed to evaluate the production of IFN-γ, IL-2, IL-4, and IL-10, and flow cytometry was performed to detect the differentiation of Th1 and Th2 subgroups.

**Results:**

lncRNA028466 was significantly decreased after the second week of immunization with r*Eg*.P29 antigen. The proportion of CD4^+^ T cells was increased after r*Eg*.P29 immunization. Overexpression of lncRNA028466 facilitated the production of IL-4, IL-10 and suppressed the production of IFN-γ, IL-2. Furthermore, after transfection with siRNA028466, IL-2 production was facilitated and IL-10 production was suppressed in naive CD4^+^ T cells.

**Conclusions:**

Immunization with r*Eg*.P29 downregulated the expression of lncRNA028466, which was related to a higher Th1 immune response and a lower Th2 immune response. Our results suggest that lncRNA028466 may be involved in r*Eg*.P29-mediated immune response by regulating cytokine expression of Th1 and Th2.

**Supplementary Information:**

The online version contains supplementary material available at 10.1186/s13071-021-04795-2.

## Background

Cystic echinococcosis (CE), also known as hydatid disease, is a worldwide chronic parasitic disease, threatening the health of livestock and humans [1]. Humans are exposed to the eggs of *Echinococcus granulosus* after close contact with infected livestock or contaminated food [2, 3]. At present, diagnosis of human echinococcosis relies mainly on imaging techniques. The life of the pathogen in the human body is relatively long, ranging from 1 to 30 years, and the symptoms of the initial infection are not obvious. Therefore, it is difficult to diagnose, treat, and control [2, 4, 5]. Due to the limitations of current diagnostic and therapeutic measures, numerous studies have been carried out in the field of immunology associated with *E. granulosus* infection in the hope of developing vaccines [6, 7]. Vaccination would be an effective preventive healthcare measure against echinococcosis [1, 8]. Due to the complicated host-parasite relationship [9], there is still no commercial vaccine.

Thus far, there have been some effective antigens as vaccine candidates for the prevention of CE. Vaccination of intermediate hosts of *E. granulosus* with the *Eg*95 antigen has induced remarkable protective efficacy [4, 10]. Another study [11] documented the P29 protein as a 29-kDa antigen, which was characterized by *E. granulosus* protoscolex-derived soluble antigen [12, 13]. Our previous studies confirmed that vaccination of r*Eg*P29 in sheep and mice carrying infection of *E. granulosus* eggs resulted in remarkable protective efficacy and induced Th1 cell to produce IFN-γ against *E. granulosus* infection [13, 14]. However, the mechanism of how the r*Eg*P29 antigen influenced the host immune responses and regulated the differentiation of CD4^+^ T cells is still unclear.

Understanding the immune response between host and parasite is essential [15]. The role of CD4^+^T cells in bacterial, viral, and parasitic infections and the adaptive immune system is important [16–18]. The distinguishing feature of the immune response caused by *E. granulosus* is that Th1- and Th2-type immune responses coexist [19]. It is commonly accepted that in most parasitic diseases, Th1 or Th2-type response is able to control pathogens; early Th1 cell activation confers host protective immunity, while Th2 cell activation is related to the progression of the chronic stage when infected with *E. granulosus* [20, 21]. The switch from a Th2 to a Th1 type of response seems to be required for a protective phenotype [22]. A polarized cytokine response plays an important role in some parasitic diseases where Th1- or Th2-type reactions are associated with susceptibility or resistance [23, 24]. Thus, understanding what impels cytokine expression toward different patterns contributes to the immune therapy and vaccination scheme [25].

lncRNAs act as pivotal regulators of gene expression in both innate and adaptive immune responses and play a crucial role in important cellular processes, like differentiation and activation of lymphocytes [26, 27]. Distinct lncRNA expression was identified from early T cells to terminal T helper (Th) cells, suggesting that lncRNAs could modulate the development and differentiation of T cells [28]. Consistent with this finding, lncRNA TMEVPG1 was specifically expressed in Th1 cells and lncR-Ccr2-50AS was proven to regulate Th2 cell differentiation [29, 30]. The ability of lncRNAs to regulate CD4^+^T cell differentiation could have crucial implications for CE, with a Th1-predominant immune response in the early infection stage and a Th2-predominant immune response in the late infection stage [31, 32]. Although lncRNAs have a variety of biological functions, the range of potential biological functions of lncRNAs in parasitic infection is vast and still largely unexplored [33, 34]. A recent study found that microRNA (miRNA), lncRNA, and circular RNA (circRNA) had been identified in protoscoleces exosome-like vesicles and hydatid fluid, and was predicted to participate in the parasite-host interactions [35]. Therefore, identifying how lncRNA regulates CD4^+^T cell differentiation could provide new mechanistic insights and therapeutic targets for CE.

We used microarray analysis to investigate the lncRNA expression profiles in splenic lymphocytes from r*Eg*.P29 immunization of mice with secondary infection of *E. granulosus*, and it showed a significant decrease in lncRNA028466 by bio-information analysis [36]. We found that after r*Eg*.P29 immunization, the expression of lncRNNA028466 in CD4^+^T cells was significantly different from other lncRNAs. We speculated that lncRNA028466 may play an important role in r*Eg*.P29-induced immune response. Therefore, lncRNA028466 was selected to further study. However, the function of lncRNA028466 has not been reported. The purpose of our study was to research the influence of lncRNA028466 on the expression of cytokines-producing Th1 and Th2 cells. Furthermore, we will look for the target molecules that interact with lncRNA028466 and further study their interactions. It is hoped that our datasets provide an important resource for future studies of the vaccine of r*Eg*.P29 and the function of lncRNAs in host-parasite interaction.

## Methods

### Mice

We obtained 6- to 8-week-old female BALB/c mice from the Animal Laboratory Center of Ningxia Medical University (SCXK2018-0004). The mice were raised in pathogen-free conditions, with eight groups of mice in each cage and fed clean food and water. All mouse experiments were permitted by the Ningxia Medical University Institutional Review Committee and carried out in strict accordance with national and institutional guidelines.

### r*Eg*.P29 antigen preparation and vaccination protocols

The recombinant plasmid was preserved in our laboratory. We then converted recombined plasmid P29/pET28a into *E. coli* *BL21* pLysS and mixed them with LB liquid medium for incubation, which induced expression at 37 °C 5 g/min for 5 h, under the condition of 1 mol/mL isopropyl-β-d-sulfur semi-lactose glycoside (IPTG, Invitrogen). Subsequently, his6-tagged r*Eg*.P29 was purified by nickel chelation affinity (Novagen) following the manufacturer’s protocol. Western blot was performed to detect the r*Eg*.P29 purity. Next, we randomly classified 24 mice into two groups of 12 mice as immune and control groups. The immune group was injected subcutaneously with 10 μg purified r*Eg*.P29 protein at three points in the abdomen. The r*Eg*.P29 protein was emulsified twice with phosphate-buffered saltwater (PBS) and Freund’s adjuvant in a volume of 100 μL. Primary immunity used Freund’s complete adjuvant, and 2 weeks after the initial immunity, Freund’s incomplete adjuvant was used to strengthen immunity. The control group was injected with an equal volume of PBS. Two weeks after the booster immunization, mice were used for later experiments.

### Western blot analysis

The BCA Protein Assay Kit (Beyotime Biotechnology) was used to measure the concentration of proteins. Then, proteins were boiled at 100 °C, 10 min, and 12% sodium dodecyl sulfate–polyacrylamide gel electrophoresis (SDS-PAGE) was used to isolate the protein. PVDF membrane (Solarbio Science & Technology, Beijing, China) was used to transfer the protein. After 2 h blockage with 5% skim milk at room temperature, the primary antibodies were supplemented at 4 °C for overnight incubation. For r*Eg*.P29 protein, the primary antibodies were immune mice and control mice serum (1:500 dilution). For total proteins, the primary antibodies included IFN-γ polyclonal antibody (rabbit anti-mouse, 1:500, cat. no. BS3486), IL-2 polyclonal antibody (rabbit anti-mouse, 1:500, cat. no. BS60299), IL-4 polyclonal antibody (rabbit anti-mouse, 1:500, cat. no. BS5764), IL-10 polyclonal antibody (rabbit anti-mouse, 1:1000, cat. no. 20850–1-AP) and β-actin monoclonal antibody-HRP (mouse anti-mouse, 1:20,000, cat. no. BS6007MH). The second antibody goat anti-rabbit IgG/HRP (1:20,000, cat. no. ZJ2020-R) was used to incubate the membrane for 2 h at room temperature. Lastly, 3,3-*N*-diaminobenzidine tetrahydrochloride horseradish peroxidase color development kit (Solarbio) was used to detect the r*Eg*.P29 protein, and electrochemiluminescence (ECL) (Tiangen, Beijing, China) was used to detect the total proteins.

### CD4^+^ T, CD8^+^ T, and B cell sorting

Following booster immunization, mice were treated by cervical dislocation. All spleen and lymphocyte operations were carried out under sterile conditions. Spleens were removed from the two groups. Subsequently, the single-cell suspension was prepared and then the spleen tissue lymphocyte isolation medium kit (Tianjin Hao Yang Biological Manufacture, Tianjin, China) was used to isolate spleen lymphocyte cells following the manufacturer’s protocol. Finally, we diluted the cell concentration to 1 × 10^7^/mL using a 10% FBS (Gibco, Grand Island, USA) PBS solution and divided it into three groups in 300 μL/tubes. Staining for cell-surface markers was performed, including APC-labeled anti-CD4 (552051), PerCP-Cy5.5-labeled anti-CD3e (562286), PE-labeled anti-CD8 (558106) FITC-labeled anti-CD19 (1575-02S) (BD Biosciences, San Jose, CA, USA), placed at 4 °C in the dark for 30 min. After washing twice, cells were suspended using 2 mL PBS with 10% FBS and sorted by flow cytometry. The purification of CD4^+^T, CD8^+^T, and B cells was > 99% (Additional file 1: Fig S1). Finally, collecting CD4^+^T, CD8^+^T, and B cells were collected for use in later experiments.

### lncRNA028466 overexpression lentivirus construction and siRNA synthesis

The endotoxin-free plasmid of lncRNA028466 was extracted using an endotoxin-free plasmid extract kit (Tiangen Biochemical Technology Co., Beijing, China) according to the manual instructions. 293 T packaging cells (Thermo Fisher Scientific, CN) were cultured with 8 mL DMEM (Invitrogen) with 10% FBS (Gibco), 1% 100 × penicillin–streptomycin solution (Invitrogen) in the condition of 37 °C and 5% CO_2_ before transfection. When the cell density reached 80–90%, 293 T cells were transfected with 9 µg pCDH-028466, 9 µg pLP1, 9 µg pLP2, and 9 µg PLP VSV-G (Invitrogen) in the presence of PolyJet (SignaGen, USA) to package the recombinant lentivirus. After 48 h of co-incubation, cells and supernatant were collected and added to a 15 mL tube at 991 g for 10 min. Then the supernatant that contained the virus was collected and filtered through a 0.45 μm filter into a new 15 mL tube. The supernatant was centrifuged at 50,000×*g* at 4 °C for 2 h. The supernatant was discarded, and the virus particles at the bottom of the tube were resuspended with 100 μL DMEM, then divided into PCR tubes and stored at −80 °C.

Small interfering RNAs (siRNAs) targeted separate sequences of lncRNA028466 were synthesized by Tianjin Sheweisi Biotechnology Company, including siRNA1, siRNA2, and siRNA3. The siRNA1 targeting sequence for lncRNA028466 is 5′-GGU UGA GAU UGG ACG UUU CAU DTD T-3′(sense) and 5′-AUG AAA CGU CCA AUC UCA ACC DTD T -3′ (antisense). The sequence of siRNA2 is 5′-GGU UGA GAU UGG ACG UUU CAU DTD T-3′ (sense) and 5′-AAA GAG GGU ACA AGG UUA GGC DTD T-3′ (antisense). The sequence of siRNA3 is 5′-GGC CAG AUA AGC UGC AAD TDT-3′ (sense) and 5′-UUG CAG CUC UUA UCU GGC CDT DG -3′ (antisense).

### Naive CD4^+^ T cell sorting, transfection, and differentiation

A healthy female BALB/c mouse spleen was placed on a 70 μm nylon cell filter to prepare a single-cell suspension. A magnetic bead isolation kit (Miltenyi Biotec, Bergisch Gladbach, Germany) was used for negatively selecting naive CD4^+^T cells following the manufacturer’s instructions. The purification of naive CD4^+^T cell populations (CD4^+^CD25^−^CD62L^hi^CD44^low^) was > 90%. Subsequently, naive CD4^+^T cells were resuspended in RPMI 1640 (Gibco, Grand Island, USA) with 2% FBS, and were cultivated at 37 °C, 5% CO_2_ in 1 × 10^6^ cells/well of 48-well plate, after which 100 μL pCDH-CMV and pCDH-028466 lentivirus or 20 nM siRNA and negative control siRNA were added in the presence of Lipofectamine 3000 (Thermo Fisher Scientific, CN); total volume was 250 μL/well. After 6 h, the transfected cells were removed into a 24-well plate. Then cells were activated with plate-bound 1 μg/mL anti-CD3 (eBioscience) plus 1 μg/mL anti-CD28, 20 ng/mL IL-2, 50 ng/mL IL-12, 10 ng/mL anti-IL-4 for Th1 conditions. For generating Th2 cells, we used 1 μg/mL anti-CD28, 20 ng/mL IL-2, 10 ng/mL IL-4, 10 ng/mL anti-IFN-γ (BD Bioscience). In the overexpression experiment, micrographs of fluorescent light were captured using a laser scanning confocal microscope. The cells were used for a later experiment after transfection for 96 h.

### Quantitative real-time PCR

Total RNA was extracted from CD4^+^T cells of the spleen using TRIzol reagent (Invitrogen) according to the manufacturer’s procedures. The total RNAs were reverse-transcribed into cDNAs by Revert Aid First Strand cDNA Synthesis Kit (Thermo Fisher Scientific, CN). PCR was accomplished on a StepOnePlus™ Real-Time PCR instrument according to specifications indicated in the Bestar™ qPCR MasterMix (SYBR Green) (DBI Bioscience). The PCR was implemented under conditions of pre-denaturation at 95 °C for 2 min, followed by 40 cycles of denaturation at 95 °C for 10 s, renaturation at 58 °C for 34 s, and extension at 72 °C for 30 s. The gene relative expression was calculated according to the 2^−ΔΔCt^ method. Specific primer sequences are listed in Table 1.

**Table 1:** Sequences of primers used for qRT-PCR

Gene	primer(5′ → 3′)
lncRNA 028466	F: AGGCCCAGTCTCTCTTGTGA R: GGGTCTGATGGGTCTCAT
IL-2	F: TGAGCAGGATGGAGAATTACAGG R: GTCCAAGTTCATCTTCTAGGCAC
IFN-γ	F: CTCCCGTGGCTTCTAGTGC R: GCCTTAGTTTGGACAGGATCTG
IL-4	F: ATCATCGGCATTTTGAACGAGG R: TGCAGCTCCATGAGAACACTA
IL-10	F: GCCACGGCACAGTCATTGA R: TGCTGATGGCCTGATTGTCTT
GAPDH	F: AGGTCGGTGTGAACGGATTTG R: GGGGTCGTTGATGGCAACA

### Flow cytometry analysis

We cultured the transfected cells under Th1 and Th2 polarizing conditions. Cell Stimulation Cocktail and Protein Transport Inhibitor (Invitrogen) were added into transfected cells in the last 6 h of incubation. For cell surface staining, we first used 0.1% BSA, 0.05% sodium azide PBS buffer to wash the collected cells. Then, the surface antibody APC-labeled anti-CD4 and PerCP-cy-5.5-labeled anti-CD3 (BD Biosciences) were used to stain the cells in the dark for 30 min at 4 °C. Subsequently, 4% paraformaldehyde was used to fix the cells under room temperature for 8 min in the dark, and 0.1% BSA, 0.05% sodium azide, and 0.1% saponin (Sigma) PBS buffer was used to permeabilize the cells at room temperature for 2 h in dark. After washing, PE-labeled anti-IFN-γ, PE-labeled anti-IL-2, PE-labeled anti-IL-4, PE-labeled anti-IL-10 (BD Biosciences) were respectively used to stain the cells in the dark for 30 min at 4 °C. Stained cells were assayed with flow cytometry (BD Biosciences) and the program FlowJo version 10.0 (Tree Star Inc. USA) was used to analyze the data.

### ELISA

Transfected cells were stimulated with Cell Stimulation Cocktail in the last 6 h of incubation. Supernatants were collected after 96 h and a specific ELISA kit (BD Biosciences, San Jose, CA, USA) was used to detect the cytokine levels following the manufacturer’s protocol.

### Statistical analysis

Using SPSS 17.0 statistical software and GraphPad Prism 6.0 software to analyze the experimental results, the measurement data were represented as mean ± standard error, according to the comparison between the two groups using the two-tailed *t*-test. The test level was *α* = 0.05.

## Results

### Microarray analysis revealed significant downregulation of lncRNA028466 transcripts in spleen lymphocytes of mice immunized with r*Eg*.P29 antigen

**Fig. 1:**
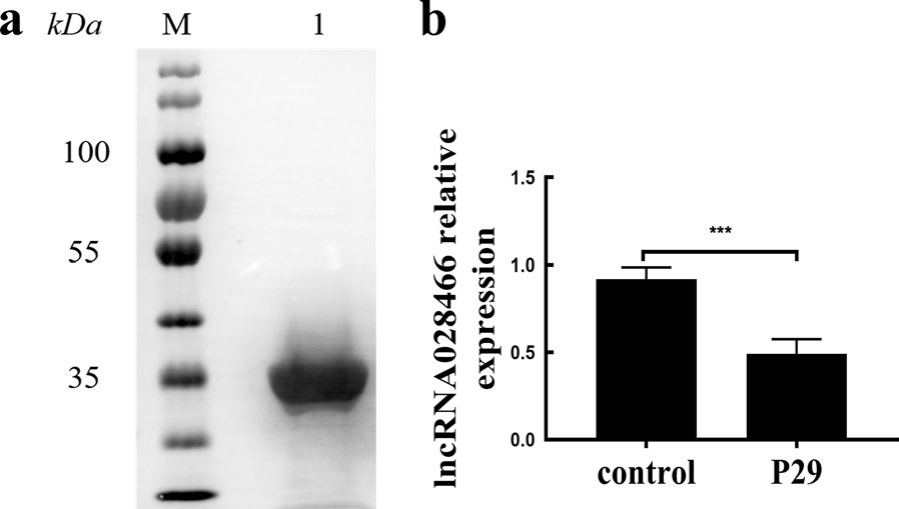
Microarray analysis reveals significant downregulation of lncRNA028466 transcripts in spleen lymphocytes of mice immunized with r*Eg*.P29 antigen. **a** r*Eg*.P29 antigen was purified and detected by western blot. **b** Validation of lncRNA028466 expression levels in lymphocytes of mice by qRT-PCR. Data were shown as mean ± SD and represented three separate experiments (*n* = 6 per group). ***P < 0.001

First, we detected a high purity expression band of r*Eg*.P29 antigen at 31 kDa through western blot (Fig. 1a). Microarray analysis revealed that lncRNA028466 was downregulated 2.31-fold in spleen lymphocytes of mice immunized with r*Eg*.P29 antigen. To verify the expression of lncRNA028466 in spleen lymphocytes following immunization, qRT-PCR was used to determine the downregulation of lncRNA028466. lncRNA028466 exhibited 1.87-fold-lower expression (*t* = 7.906, *P* = 0.0002) compared with that in the control group (Fig. 1b).

### The expression of lncRNA028466 was decreased in CD4^+^ T cells of spleens from mice immunized with r*Eg*.P29 antigen

**Fig. 2:**
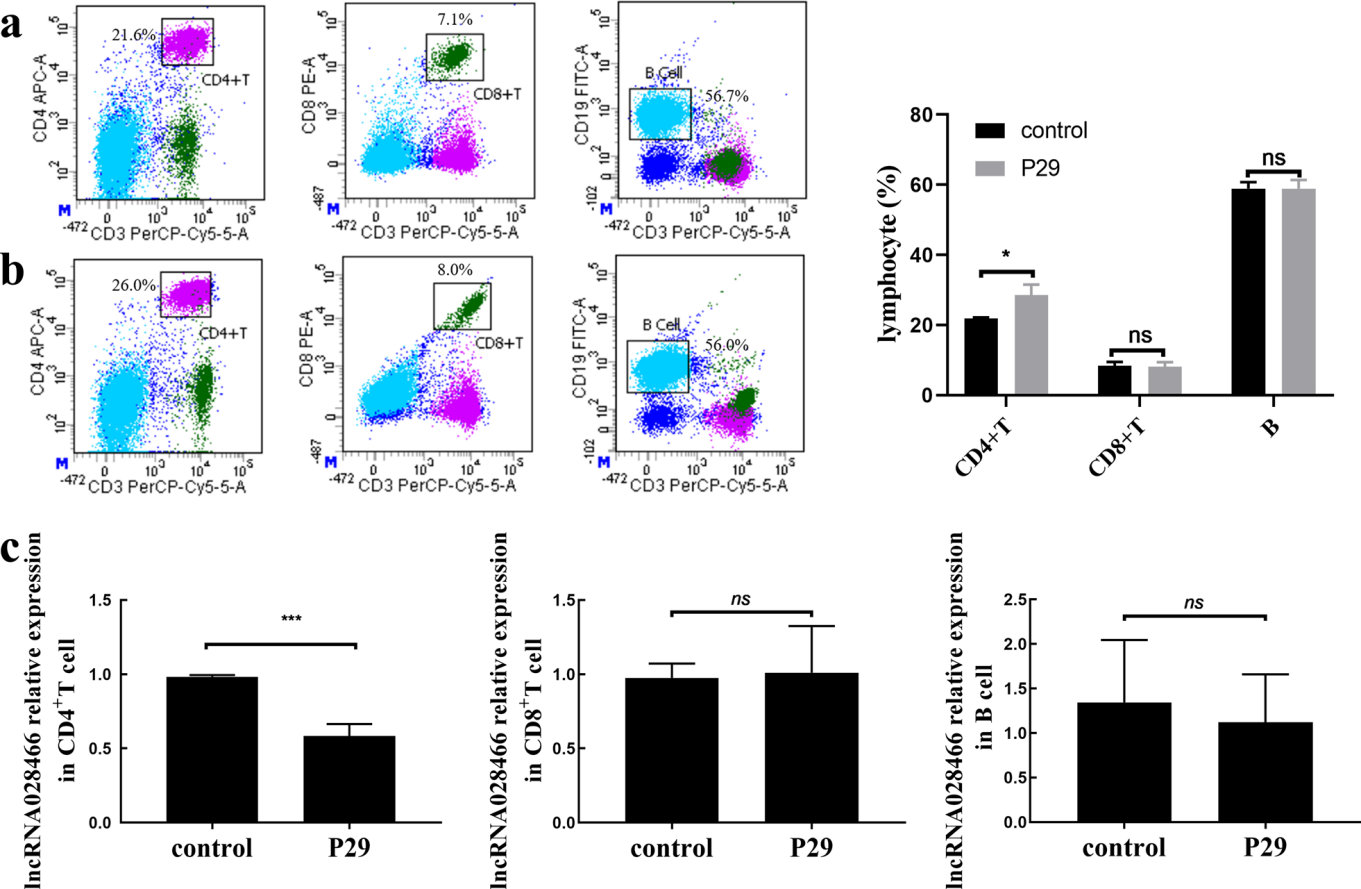
The expression of lncRNA028466 was decreased in CD4^+^T of spleens from mice immunized with r*Eg*.P29 antigen. Following boost immunization, flow cytometry was used to isolate CD4^+^T, CD8^+^T and B cells. The percentage of CD4^+^T, CD8^+^T and B cells in the control (**a**) and immune groups (**b**) are shown. **c** The expression of lncRNA028466 in CD4^+^T, CD8^+^T and B cells was measured by qRT-PCR. Results are shown as mean ± SD and represent three separate experiments (*n* = 6 per group). *P < 0.05, **P < 0.01, ns: not significant

Because lncRNA028466 expression decreased in spleen lymphocytes of mice immunized with r*Eg*.P29 antigen, we aimed to understand the role that lncRNA028466 played in r*Eg*.P29-mediated immune response. We then respectively isolated splenic CD4^+^T, CD8^+^T, and B cells from the control and immune groups using flow cytometry. The percentage of CD4^+^T, CD8^+^T, and B cells were respectively 21.6%,7.1%, 56.7% in the control group (Fig. 2a) and 26%, 8.0%, 56.0% in the immune group (Fig. 2b). The data indicated that CD4^+^T cells were increased following immunization with r*Eg*.P29. qRT-PCR was performed to measure the expression of lncRNA028466 in CD4^+^T, CD8^+^T, and B cells. The expression of lncRNA028466 in CD4^+^T cells from the immune group was 1.68 times lower (*t* = 9.651, *P* < 0.001) than the control group; however, there were no prominent changes in CD8^+^T and B cells (Fig. 2c). All in all, these data suggested that lncRNA028466 could affect cellular immune responses associated with r*Eg*.P29 antigen immunity.

### Overexpression of lncRNA028466 biased naive CD4^+^ T cell differentiation toward the Th2 phenotype

**Fig. 3:**
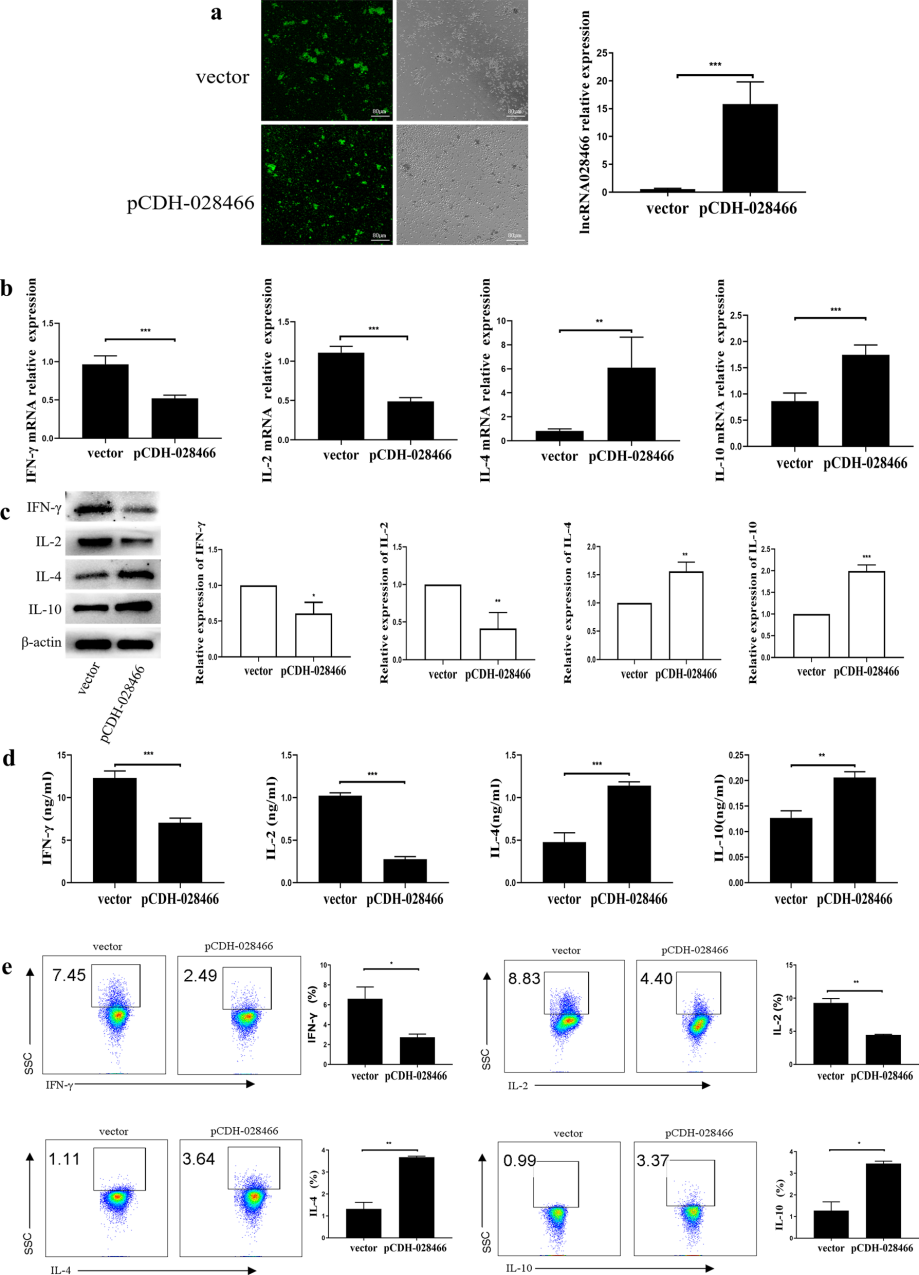
Overexpression of lncRNA028466 biases naive CD4^+^T cell differentiation toward the Th2 phenotype. **a** Validation of the transfection efficiency of lncRNA028466 overexpression lentivirus by qRT-PCR and representative image of the expression of GFP (×40) was detected by a laser scanning confocal microscope (Olympus). **b** The mRNA expression of IFN-γ, IL-2, IL-4, and IL-10 was measured by qRT-PCR. **c** The protein levels of cytokines were determined by western blot. **d** The supernatants were detected by ELISA. **e** The differentiation of the transfected naive CD4^+^T cells was measured by FACS. The scattered dots in the box represent the expression of IFN-γ and IL-2 producing Th1 cells, and IL-4 and IL-10 producing Th2 cells. Results are shown as mean ± SD and represent three separate experiments (*n* = 6 per group). Vector represents empty vector, pCDH-028466 represents lncRNA028466 overexpression vector. *P < 0.05, **P < 0.01, ***P < 0.001

To understand the role that lncRNA028466 played in CD4^+^T cell differentiation, we isolated splenic naive CD4^+^T lymphocytes. In addition, we used 293 T cells packing lncRNA028466 overexpression lentivirus (pCDH-028466, green fluorescent protein [GFP]) and empty vector (pCDH-CMV, GFP). The expression of GFP was detected by a laser scanning confocal microscope (Olympus) (Fig. 3a). Then, the transfection efficiency of transfection naive CD4^+^ T cells with pCDH-028466 and pCDH-CMV was detected by qRT-PCR analysis. lncRNA028466 overexpression lentivirus exhibited a 28.56-fold-higher expression (*t* = 7.924, *P* = 0.0005) compared with empty vector (Fig. 3a). The result suggested that the lncRNA028466 overexpression lentiviral vector was successfully transfected into the cells. Subsequently, naive CD4^+^T cells were infected with pCDH-CMV and pCDH-028466. Then, the expression of Th1-related cytokines IL-2, IFN-γ, and Th2-related cytokines IL-10, IL-4 were detected respectively from mRNA, protein, and extracellular levels by qRT-PCR, western blot and ELISA. As a result, according to qRT-PCR analysis, overexpression of lncRNA028466 inhibited the expression of IFN-γ (*t* = 7.564, *P* = 0.0003) and IL-2 (*t* = 13.28, *P* < 0.001) and increased the expression of IL-4 (*t* = 4.157, *P* = 0.006) and IL-10 (*t* = 7.367, *P* = 0.0003) (Fig. 3b). The result was confirmed by western blot and ELISA analysis (Fig. 3c, d).

Considering the result that overexpression of lncRNA028466 upregulated Th2-related cytokines IL-4 and IL-10 expression, and downregulated Th1-related cytokines IFN-γ and IL-2 expression, we studied the role of lncRNA028466 in CD4^+^T cell differentiation. pCDH-CMV and pCDH-028466 were transfected into naive CD4^+^T cells, which were cultured in the Th1 and Th2 cell–polarizing conditions. The FACS analysis showed that lncRNA028466 overexpression of lentivirus-transfected cells increased differentiation toward IL-4- and IL-10- (Fig. 3e) producing Th2 subtype, compared with the empty vector group. However, there was a remarkable reduction in IFN-γ- and IL-2-producing (Fig. 3e) Th1 cells. All in all, these results may indicate that overexpression of lncRNA028466 biases naive CD4^+^T cell differentiation toward the Th2 phenotype.

### Knockdown of lncRNA028466 promotes naive CD4^+^ T cell differentiation toward the Th1 subgroup

**Fig. 4:**
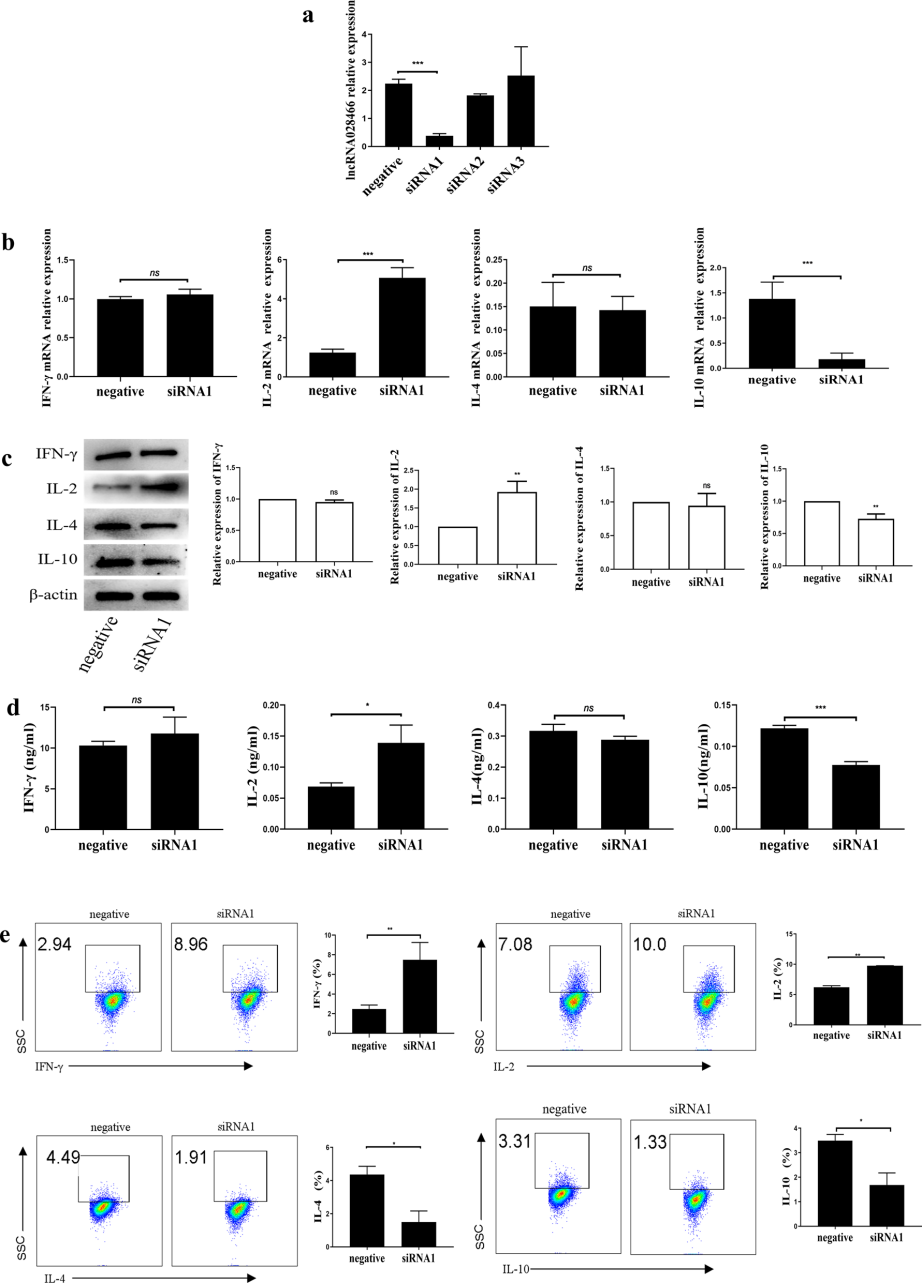
Downregulation of lncRNA028466 promotes naive CD4^+^T cell differentiation toward the Th1 phenotype. **a** The interference efficiency of siRNAs was tested by qRT-PCR. **b** The mRNA expression of cytokines in the transfected naive CD4^+^T cells was tested by qRT-PCR. **c** The protein levels of cytokines were determined by western blot. **d** The supernatants were detected by ELISA. **e** The differentiation of the transfected naive CD4^+^T cells was measured by FACS. The scattered dots in the box represent the expression of IFN-γ and IL-2 producing Th1 cells, and IL-4, and IL-10 producing Th2 cells. The data came from a single experiment and represented three separate experiments. Negative represented the control group, siRNA1 represented the lncRNA028466 knockdown group. Data were expressed as mean ± SD and represented three separate experiments (*n* = 6 per group). *P < 0.05, **P < 0.01, ***P < 0.001, ns: not significant

To confirm whether lncRNA028466 contributes to CD4^+^T cell differentiation, we designed siRNA to knock down its expression in naive CD4^+^T cells. For siRNA interference efficiency, siRNA1, siRNA2, and siRNA3 were respectively transfected into naive CD4^+^T cells, and qRT-PCR was performed. According to the result (Fig. 4a), siRNA1 efficiently knocked down lncRNA028466 by 5.88-fold (*t* = 17.98, *P* < 0.001) compared with negative siRNA and was used for later experiments. Subsequently, siRNA1 and negative siRNA were transfected into naive CD4^+^T cells, and qRT-PCR, western blot, and ELISA were performed to measure the expression of Th1-related cytokines IL-2, IFN-γ, and Th2-related cytokines IL-10, IL-4. We found that knockdown of lncRNA028466 upregulated IL-2 (*t* = 14.10, *P* < 0.0001) production and downregulated IL-10 (*t* = 6.822, *P* = 0.0005) production (Fig. 4b–d). There was a mild increase in IFN-γ, and a mild decrease in IL-4 (Fig. 4b–d); however, the levels of IFN-γ and IL-4 did not reach statistical significance. The low levels may have gone undetected.

Similarly, siRNA-transfected naive CD4^+^T cells were placed in the Th1 and Th2 cell–polarizing conditions. The FACS analysis showed that siRNA-transfected cells differentiated toward IFN-γ- and IL-2-producing Th1 subtype (Fig. 4e), compared with a negative control group. However, there was a remarkable reduction in the Th2-related cytokines IL-4 and IL-10 (Fig. 4e). Taken together, the results indicate that knockdown of lncRNA028466 promotes naive CD4^+^T cell differentiation toward Th1 cells.

## Discussion

The purpose of our study was to research the contribution of lncRNA028466 to the differentiation of splenic CD4^+^T cells. CD4^+^T cells are major participants in CE, and the bias of CD4^+^T cells toward the Th2 subset causes an unbalanced secretion of cytokines producing Th subsets, which results in the chronic progression of CE [37, 38]. Consistent with this theory, in our study, the proportion of CD4^+^T cells was augmented following immunization with r*Eg*.P29. This meant that CD4^+^T cells participate in the immunoreaction associated with r*Eg*.P29.

The expression of lncRNA was lineage-specific and played a crucial role during CD4^+^T cell differentiation toward Th1 and Th2 cells [28, 39, 40]. At present, the relevance of lncRNA to the pathogenesis, host resistance, and parasite evasion during parasitic infection is emphasized [34]. There is increasing attention to the contribution of lncRNA to *E. granulosus* infection [35]. This kind of research will contribute to the reasonable design of immune therapy and vaccination schemes. In the present study, we discovered that lncRNA028466 significantly decreased in CD4^+^T cells with the r*Eg*.P29 immunized group. On the one hand, overexpression of lncRNA028466 not only enhanced differentiation of Th2 cells but also promoted the secretion of Th2-related cytokines IL-10 and IL-4, but suppressed the expression of Th1-related cytokines IFN-γ and IL-2. On the other side, the knockdown of lncRNA028466 inhibited the differentiation of Th2 cells and promoted the differentiation of Th1 cells. Based on this finding, we propose that lncRNA028466 may participate in the protective r*Eg*.P29-mediated immunity by modulating Th1 and Th2 cytokine expression.

The polarization of the Th1 and Th2 subset is a crucial determinant of whether the response to the pathogen will confer protection to the host or exacerbate the disease [24]. IFN-γ is demonstrated to inhibit *Echinococcus* activity and contributes to the formation of the protective immunity mediated by Th1 cells during *E. granulosus* infection [25, 38]. Mouse experimental studies supporting IL-4- and IL-10-mediated immunosuppression may be key to parasite evasion and survival [19, 41]. This study demonstrated that the increased Th1 immune response was related to lower IL-4 and IL-10 expression, as well as lncRNA028466 overexpression. The immunoprotection of r*Eg*.P29 may be realized by lncRNA028466 regulating the expression of cytokines associated with Th1 and Th2 cells. However, our study just preliminary investigated the role of lncRNA028466 in regulating the expression of Th1- and Th2-associated cytokines in vitro. To further demonstrate the result, we will further study the function of lncRNA028466 in vivo and in vitro.

## Conclusions

In conclusion, lncRNA028466 may be involved in r*Eg*.P29 vaccination-mediated Th1 protective immunity. The study of the function of lncRNA in T helper responses is an area that needs to be explored, which will help deepen our learning about the complicated immune response. We expect that further characterization of the identified lncRNA028466 will reveal the important role of lncRNA in host-parasite interaction and CD4^+^ T cell differentiation.

## Supplementary Information

**Additional file 1: Figure S1**. The purification of CD4^+^T, CD8^+^T, and B cells. CD4^+^T, CD8^+^T, and B cells from spleen of mice immunized with r*Eg*.P29 antigen were sorted by flow cytometry. **a** The purification of CD4^+^T cells. **b** The purification of CD8^+^T cells. **c** The purification of B cells.

## Data Availability

The datasets supporting the findings of this article are included within the paper. The study-related data can be obtained from the corresponding author upon request.

## Abbreviations

Eg: *Echinococcus granulosus*
lncRNA: Long noncoding RNA
r*Eg*.P29: Recombinant *Echinococcus granulosus* antigen P29
PBS: Phosphate-buffered solution
IPTG: Isopropyl β-d-thiogalactoside
FBS: Fetal bovine serum
DMEM: Dulbecco's modified Eagle’s medium
RPMI1640: Roswell Park Memorial Institute
qRT-PCR: Quantitative real-time polymerase chain reaction
Treg: Natural regulatory cell
BSA: Bovine serum albumin
