# Supplementary figures and images for: lncRNA028466 regulates Th1/Th2 cytokine expression and associates with Echinococcus granulosus antigen P29 immunity
Source: Parasit Vectors. 2021 Jun 3;14:295. doi: 10.1186/s13071-021-04795-2 (PMC8173744; doi:10.1186/s13071-021-04795-2)

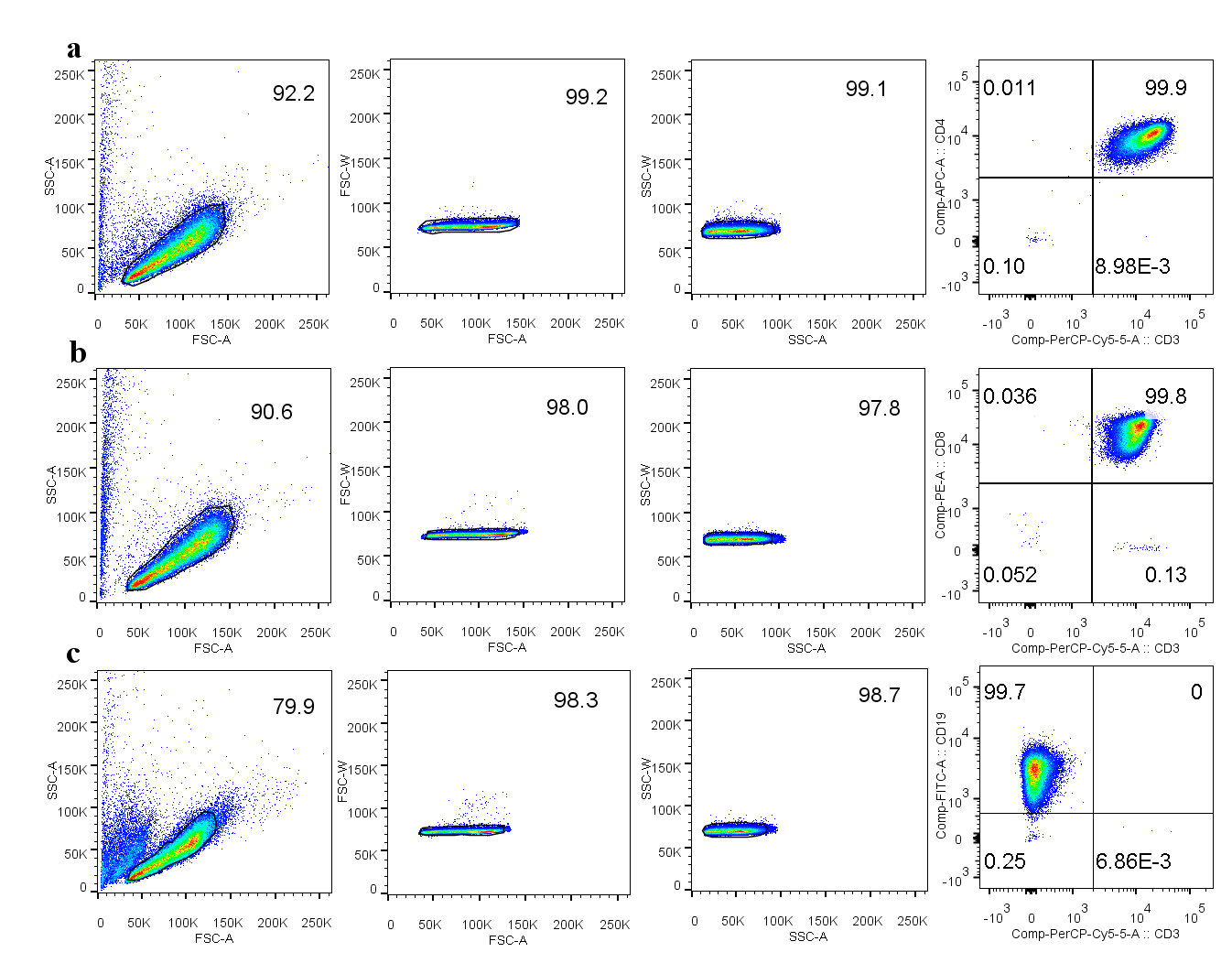

Supplement: Supplementary file 1 — Additional file 1: Figure S1. The purification of CD4+T, CD8+T, and B cells. CD4+T, CD8+T, and B cells from spleen of mice immunized with rEg.P29 antigen were sorted by flow cytometry. a The purification of CD4+T cells. b The purification of CD8+T cells. c The purification of B cells. [file 13071_2021_4795_MOESM1_ESM.png]
